# Co-cultures of Glioma Stem Cells and Primary Neurons, Astrocytes, Microglia, and Endothelial Cells for Investigation of Intercellular Communication in the Brain

**DOI:** 10.3389/fnins.2019.00361

**Published:** 2019-04-18

**Authors:** Zhiyun Wei, Shubham Kale, Rachid El Fatimy, Rosalia Rabinovsky, Anna M. Krichevsky

**Affiliations:** Department of Neurology, Brigham and Women’s Hospital, Harvard Medical School, Boston, MA, United States

**Keywords:** intercellular communication, extracellular vesicles, glioma stem cells, neurons, microglia, astrocytes

## Abstract

Intercellular communication within complex biological and pathological systems via extracellular vesicles (EVs) and secreted factors is a highly attractive area of research. However, cell models enabling investigation of such communication *in vitro* are limited. Commonly utilized is the supplementation of hyper-concentrated EVs or other extracellular factors to the recipient cell cultures. This approach requires purification of the secreted complexes and is confounded by the contamination of media components. Two-chamber co-cultures of donor and recipient cells separated by a pore membrane may represent a more physiological and better-controlled system for the investigation of intercellular communication. Yet, distinct culture conditions for different neural cell types often make them incompatible for co-culturing. Here we optimized short-term co-cultures of patient-derived low-passage glioma-initiating stem cells with normal cells of the brain microenvironment, such as primary neurons, astrocytes, microglia, and brain endothelial cells. We demonstrate the culture compatibility of these cell types and internalization of glioma-derived extracellular RNA by the normal recipient cells. The presented protocols are valuable for the investigation of intercellular communication between glioma brain tumor and cells of its microenvironment, including but not limited to the EVs-mediated communication.

**RESEARCH IN CONTEXT:**

Cell-to-cell communication is essential in normal physiology and implicated in disease; however, experimental systems for its modeling *in vitro* are limited. Particularly, the investigation of communication between brain tumors and normal cells of the brain microenvironment has been challenged by the lack of adequate culture models. Here we developed co-cultures of glioma stem cells with various types of normal brain cells, including primary neurons, astrocytes, microglia, and brain endothelial cells, and demonstrated their utility for the study of intercellular communication. Detection of proposed markers in the recipient cells confirmed RNA transfer in these co-cultures.

## Highlights

- Culture conditions were optimized for co-culturing glioma stem cells with various types of primary normal brain cells.
- RNA transfer between donor and recipient cells was confirmed in the co-cultures.

## Introduction

Intercellular communication is critical for all biological and pathological processes. Established secreted mediators of such communication include hormones, neurotransmitters, growth factors, cytokines, chemokines, and neurotrophins. More recently, additional routes of transmission, such as those mediated by extracellular vesicles (EVs) (Valadi et al., 2007; Skog et al., 2008) and extravesicular ribonucleoproteins (RNPs) (Vickers et al., 2011) carrying RNA, proteins, lipids and other cargo molecules, have also been extensively explored.

Investigation of these modes of communication is an especially active area of research in the field of cancer biology, as they are thought to contribute to the invasion, metastasis, angiogenesis, chemo-resistance, and immune tolerance of many malignancies (Hanahan and Weinberg, 2011). Among these malignancies is glioblastoma (GBM), the most common and invasive primary brain tumor that affects both adult and pediatric patients. The median survival of GBM patients is only about 14–15 months, despite aggressive treatments that include tumor resection followed by radiotherapy and chemotherapy. Investigation of the tumor cross-talk with other cells of the brain microenvironment may provide new avenues for the development of targeted therapeutic applications (reviewed by Broekman et al., 2018).

The most common approaches for the investigation of the intercellular communication rely on either a single-pulse or repeated supplementation of the purified and concentrated “agent” (e.g., EVs) to the recipient cells. However, such a strategy does not consider the local physiological concentration and the dynamics of release and uptake of the investigated factors. Another way of studying intercellular communication employs co-cultures of donor and recipient cells in common culture conditions, with a membrane separating the two cell types. Comparing to the previous strategy, co-culture experiments may better model the complex and persistent communication between the cells, physiological release-uptake dynamics, and enable assessment of multiple mediators of communication between the donor and recipient cultures. Such experimental design has been utilized in many studies, including those reporting miRNA transfer from cardiac fibroblast to cardiomyocytes (Bang et al., 2014) and between dendritic cells (Alexander et al., 2105), ncRNA transfer from cardiosphere-derived cells to macrophages (Cambier et al., 2107), mRNA transfer from acute myelogenous leukemia to stromal niche (Huan et al., 2103), DNA transfer between parasites (Regev-Rudzki et al., 2013), protein transfer from mesenchymal stromal cells to haematopoietic cells (Bauer et al., 2011), and virus transfer from infected hepatoma cells to plasmacytoid dendritic cells (Feng et al., 2014). In these cases, both donor and recipient cells share the same, or similar, routine culturing condition and require no additional optimization for co-culturing. However, the studies of intercellular communication in the brain and brain tumors are largely hampered by the difficulty in co-culturing glioma with primary neuroglial cells, as they have distinct culture conditions.

Here we screened multiple conditions and developed novel protocols for co-culturing patient-derived glioma-initiating stem cells (GSC), a key population of GBM cells that is considered most resistant to chemo- and radiotherapies and largely responsible for tumor recurrence, with primary neuronal, glial, and endothelial cells of the brain. We provide two new species-specific ncRNA markers to demonstrate extracellular RNA (exRNA) transfer from GSC to the normal cells. The established cell systems may provide a simple and physiologically relevant model for studying the bidirectional communication in malignant gliomas.

## Materials and Methods

### Human GSC Cultures

Low passage GBM8 cells have been cultured as neurospheres in Neurobasal medium (Gibco) supplemented with 3 mM GlutaMAX (Gibco), 1× B-27 supplement (an optimized serum-free proprietary supplement commercially available from Gibco), 0.5× N-2 supplement (a chemically defined, serum-free supplement from Gibco; components include transferrin, insulin, progesterone, putrescine and selenite), 0.5% Antibiotic-Antimycotic Solution (Corning), 20 ng/mL EGF (R&D Systems, MN, United States), and 20 ng/mL FGF (PeproTech, NJ, United States). Mature neurospheres were dissociated using NeuroCult Chemical Dissociation Kit (Mouse) (Stemcell Technologies, Canada). Approximately 0.5 × 10^6^ cells were seeded in 10 mL fresh media in a 10 cm dish (Corning), and 1/3 volume of fresh medium was added every 3 days. Mature neurospheres were usually formed within 7∼10 days.

### Primary Neuron Cultures

Brain cortical tissues of E18 C57BL/6 mice were dissected, dissociated with papain (12 U/ml; Worthington) for 20 min at 37°C, and triturated to single cells using Pasteur pipettes. After dissociation, the cells were washed with DMEM-F12 (Corning) three times and pelleted at 300 *g* centrifugation. The cells were seeded on a poly-D-lysine (Sigma-Aldrich, MO, United States) coated 24-well plates, at 80,000 cells per cm^2^. The seeding medium consisted of Neurobasal, 1× B-27, 1% Antibiotic-Antimycotic Solution (Corning), 0.5 mM GlutaMAX, and 2% FBS (Gibco). Next day, the seeding medium was replaced to the fresh culture medium with no FBS. After 5 days, the culture medium was supplemented with Ara-C (5 μM; Sigma-Aldrich) to deplete glial cells. Half of the medium was replaced with the fresh culture medium twice a week.

### Primary Glia Cultures

Brain cortical tissues of P1 C57BL/6 mice were cut to small pieces and dissociated with 0.25% Trypsin (Gibco) and 0.1 mg/mL DNase I (Roche) for 15 min at 37°C, with swirling every 3 min. After dissociation, the cells were washed with DMEM-F12 three times and pelleted at 300 *g* centrifugation. The cells collected from three pups were seeded in one T75 flask with culture medium consisted of DMEM-F12, 10% FBS, and 1% Antibiotic-Antimycotic Solution. The medium was changed to the fresh culture medium 3 days after seeding, and further replaced every 5–7 days. For astrocyte cultures, the flasks were shaken at 200 rpm at 37°C overnight three times to remove microglia, and then trypsinized and transferred to 24-well plates. For microglia cultures, the media was supplemented with a recombinant M-CSF mouse protein (10 ng/mL; Gibco). One week later, floating microglia were collected by pelleting the conditioned media at 300 g for 10 min, and then seeded on the poly-D-lysine coated 24-well plates, at 100,000 cells per cm^2^ with fresh culture media.

### Cultures of Brain Endothelial Cells

Mouse primary brain microvascular endothelial cells (MBEC) were purchased from Cell Biologics, IL, United States (Catalog# C57-6023; Lot# 070613T2MP) and cultured according to the manual, but with no heparin (as it interferes with EV uptake Atai et al., 2013; Christianson et al., 2013). Briefly, T25 flasks were pre-coated with Gelatin-based coating solution (Cell Biologics), 10^6^ cells seeded in the Endothelial Cell Medium (Cell Biologics) and passaged 1:2 upon confluence. Low passages (1–4) have been used in this study.

### Co-culture Conditions

The recipient normal cells of the brain were seeded in 24-well plates, as described above. One day later, small GBM8 neurospheres grown in GSC conditions were transferred to the upper chamber of the Millicell hanging insert with 1.0 μm pore size (Millipore). For co-cultures of GSCs with neurons, the optimized media consisted of Neurobasal, 1× B-27, 0.5× N-2 supplement, 0.5 mM GlutaMAX, and 1% Antibiotic-Antimycotic Solution. For co-cultures of GSCs with glia, the optimized media consisted of DMEM-F12, 1× B-27, 0.5× N-2 supplement, and 1% Antibiotic-Antimycotic Solution. For co-cultures of GSCs with endothelial cells, the optimized media consisted of Mouse Endothelial Cell Basal Medium (Cell Biologics), 1× B-27, 0.5× N-2, 0.1% VEGF, 0.1% ECGS, 0.1% EGF, 0.1% Hydrocortisone, 2 mM L-Glutamine and 1% Antibiotic-Antimycotic Solution.

### Cell Imaging

Using the IN Cell Analyzer 2200 (GE Healthcare Life Sciences, PA, United States) the 24-well plates were automatically scanned with 81 photographs per well taken at days 3 and 6 of cell culturing in the experimental media. The representative images of neurospheres were adjusted by auto-contrast, and for adherent monolayer cultures, additionally, by Scott 5 HDR preset of the Photoshop (Adobe, CA, United States). All images have been processed in parallel, using identical settings.

### RNA Isolation and qRT-PCR

Total RNA was isolated by miRCURY RNA Isolation Kit – Cell & Plant (Exiqon, Denmark), with on-column DNase treatment (Qiagen, Germany), after 3 days of co-culture. RNA quality was examined using Agilent 2100 Bioanalyzer (Agilent, CA, United States). qRT-PCR reactions were performed using miRCURY system (Exiqon) as previously reported (Wei et al., 2017) for small RNAs, and iScript system (Bio-Rad, CA, United States) for mRNAs, with the following DNA primers. Human RNY5 RNA (Christov et al., 2006): 5′-AGTTGGTCCGAGTGTTGTGG-3′ and 5′-AACAGCAAGCTAGTCAAGCG-3′; human RNU2-1 RNA: 5′-TTTGGAGCAGGGAGATG-3′ and 5′-CACCGTTCCTGGAGGTA-3′; mouse Actb: 5′-GGCACCACACCTTCTACAATG-3′ and 5′-GGTACGACCAGAGGCATACA-3′; mouse Gapdh: 5′-GAAGGTCGGTGTGAACGGATT-3′ and 5′-CGTGAGTGGAGTCATACTGGAAC-3′; mouse NeuN: 5′-GCACGGCATGACCCTCTACA-3′ and 5′-GTGGAGTTGCTGGTTGTCTGTC-3′; mouse Tuj1: 5′-CAGCGATGAGCACGGCATAGA-3′ and 5′-CCAGGTTCCAAGTCCACCAGAAT-3′; mouse Gfap: 5′-AACGACTATCGCCGCCAACTG-3′ and 5′-CGAGCAAGTGCCTCCTGGTAAC-3′; mouse Eaat1: 5′-TGCCTCTCCTCTACTTCCTG-3′ and 5′-CCACACCATTGTTCTCTTCCA-3′; mouse CD11b: 5′-AGCACCTCGGTATCAGCATATTG-3′ and 5′-GGTATTGCCATCAGCGTCCAT-3′; mouse Iba1: 5′-CAGACTGCCAGCCTAAGACA-3′ and 5′-GGATCATCGAGGAATTGCTTGT-3′; mouse CD31: 5′-TCCAACAGAGCCAGCAGTATGA-3′ and 5′-GCGATGACCACTCCAATGACAA-3′; mouse Cdh5: 5′-CCAGCGACACTTCTACCACTTC-3′ and 5′-CTGTCACTGGTCTTGCGGATG-3′. The U6 snRNA was quantified with LNA-containing primers (Exiqon) and used as the endogenous control for small RNAs.

### Western Blot

Cell lysates containing equal amount of total proteins were separated by SDS-PAGE with 4–12% Blot Bis-Tris Plus Gels (Thermo Fisher Scientific), and transferred to 0.45 μm PVDF membrane (Thermo Fisher Scientific). After blocking with 5% (wt/vol) fat-free milk in Tris-buffered saline with 0.075% Tween-20 (TBST), the membranes were incubated overnight with diluted primary antibodies (Tuj-1: Abcam ab14545, 1:10000 dilution; GFAP: Sigma-Aldrich G9269, 1:7500 dilution; CD11b: Abcam ab133357, 1:2000 dilution; CD31: Cell Signaling Technology 3528S, 1:500 dilution; β-Actin: Abcam ab3280, 1:10000 dilution) at 4°C. The membranes were washed and incubated with horseradish peroxidase-conjugated secondary antibodies (Cell Signaling Technology 7074S and 7076S, 1:5000 dilution) for 1 h at room temperature. The blots were developed by the Amersham ECL Reagent (GE Healthcare).

## Results

### GSCs Cultured in Alternative Conditions Change Their Morphology

For maintenance of the stem-cell-like properties, GSCs are typically cultured in a defined serum-free Neurobasal media, whereas primary astrocytes, microglia, and endothelial cells are cultured in distinct growth conditions, supplemented with FBS. Optimal growth conditions for GSCs and primary neurons are more similar, with the supplementation of N-2 and additional growth factors in GSC media, which suggest their better compatibility in the co-cultures.

**FIGURE 1:**
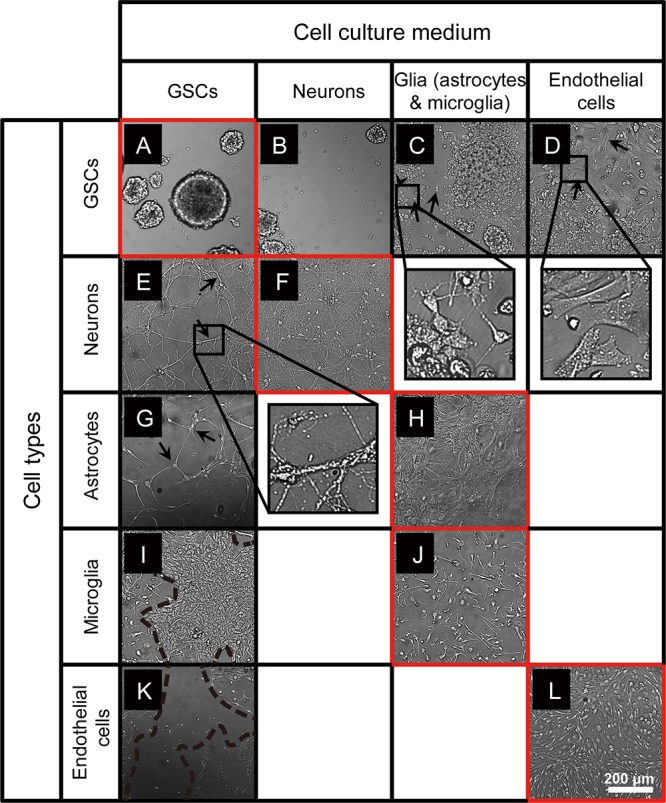
Microscopy images of GSCs, primary neurons, astrocytes, microglia, and endothelial cells in various culture conditions. **(A–D)** Typical GBM8 spheroids cultured in GSC medium **(A)**, and their altered growth in neuron medium **(B)**, glia medium (**C**; alterations observed in 81 out of 81 images analyzed), and endothelial cell medium (**D**; observed in 81 out of 81 images analyzed). **(E,F)** Representative images of primary neurons cultured in regular medium **(F)** and their morphological changes in GSC medium (**E**; observed in 17 out of 81 images analyzed). **(G,H)** Representative images of primary astrocytes cultured in regular medium **(H)** and their morphological changes in GSC medium (**G**; observed in 5 out of 81 images). **(I,J)** Representative images of primary microglia cultured in regular medium **(J)** and their morphological changes in GSC medium (**I**; observed in 30 out of 81 images). **(K,L)** Representative images of primary brain endothelial cells cultured in regular medium **(L)** and their morphological changes in GSC medium (**K**; observed in 78 out of 81 images). Typical morphological alterations are depicted by arrows, arrowhead, and dashed lines. Images were taken 6 days after medium change. *N* = at least two independent experiments/condition.

To investigate whether GSCs can maintain their morphology in conditions used for culturing normal cells of the brain, we established small spheroids of GBM8 GSCs, and at day 3 after passaging, replaced the GSC growth medium with the media corresponding to either neuronal, glial, or endothelial cells, followed by the observation of GSC morphology over 6 days. As shown in Figure 1A, when cultured in the regular GSC medium, in 9 days GSCs grow into spheroids of variable sizes. Similar, albeit smaller GSC spheroids are formed in the neuronal media lacking N-2 supplements (Figure 1B). However, as expected, when cultured in the FBS-containing media used for glial or endothelial cells, small gliomaspheres became adherent to the plate surface and exhibit altered morphology, indicative of their differentiation (Figure 1C,D). Most of the cells spread out of the initial spheres to form monolayer or multilayer colonies. In the glial medium that is essentially similar to the media used for GSC differentiation, GSCs developed multiple extended processes (arrows in Figure 1C) with frequent branching (arrowhead in Figure 1C), resembling the premature primary astrocytes (observed in 81 out of 81 images analyzed). In the endothelial medium, GSCs exhibit fibroblastic morphology with bipolar or multipolar elongated shapes (arrows in Figure 1D; observed in 81 out of 81 images analyzed).

### Primary Brain Cells Change Their Morphology When Cultured in GSC Media

To examine the compatibility of primary neurons, astrocytes, microglia, and endothelial cells with GSC medium, we established individual cultures of these cells using their optimal growth conditions, then replaced the media with the GSC medium, and observed their growth over 6 days. Primary neuronal cultures grown in the GSC media at DIV10 to 16 exhibited altered cell morphology and reduced complexity, with fewer and less branched processes (arrows in Figure 1E, compared to Figure 1F).

Primary astrocytes usually maintain their morphology and remain adherent and healthy for several months, as shown in Figure 1H. However, when the pre-established monolayer of astrocytes was exposed to the GSC medium, the cells dramatically changed their shape, become slender and more elongated, and the cultures lost their typical dense monolayer appearance (arrows in Figure 1G).

Microglia cultured in the established optimal conditions is firmly attached to the polylysine matrix, displays a typical ramified morphology, and remains quiescent (Figure 1J). However, when cultured in the GSC media, microglia became activated and proliferative, giving rise to large colonies (area surrounded by dashed lines in Figure 1I).

Finally, the cultures of primary brain endothelial cells (Figure 1L) exhibited significant cell death and quickly degenerated when grown in the GSC medium (Figure 1K). Taken together, these results indicate that the culture conditions utilized for GSCs and normal primary cells of the brain are incompatible and cannot be for co-culture experiments.

### Optimization of Co-culture Conditions for GSC and Primary Normal Cells of the Brain

**FIGURE 2:**
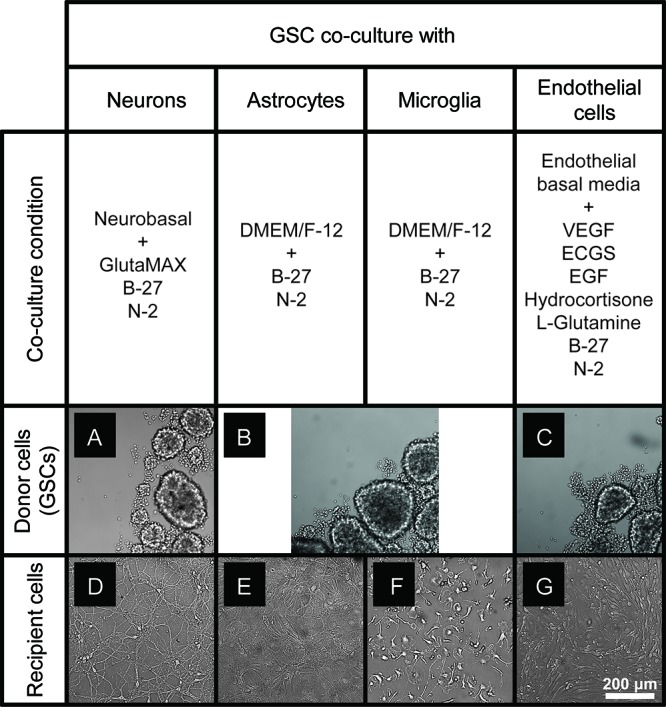
GSC and normal cell types of the brain maintained in the optimized co-culture conditions for 6 days. **(A–C)** GBM8 spheroids in co-culture media optimized for neurons **(A)**, glia **(B)**, and endothelial cells **(C)**. **(D–G)** Representative images of primary neurons **(D)**, astrocytes **(E)**, microglia **(F)**, and brain endothelial cells **(G)** in the corresponding co-culture media. *N* = at least two independent experiments/condition.

**FIGURE 3:**
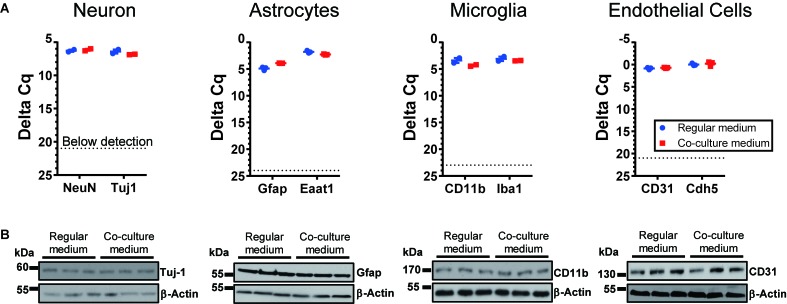
Expression of cell-type specific markers for cells cultured under regular conditions and the optimized co-culture conditions. No significant difference was observed, based on RNA **(A)** and protein levels **(B)**. *N* = 3 wells per culture condition.

To optimize the GSC co-cultures with normal cells of the brain, for each pair of cell types we tested several media compositions based on their original recipes (Supplementary Table S1) and observed the cell growth and morphology over time. The co-culture conditions were optimized to enable the maintenance of the undifferentiated neurosphere state by GSCs (Figure 2A–C) and preserve the original morphologies of the counterpart normal cells (Figure 2D–G) for at least 6 days. Loss of neuronal processes has been rarely observed in the optimized conditions (in only 4/81 images in the co-culture conditions, which is similar to 3/81 rate observed in normal neuronal cultures). Abnormal morphologies were not observed for astrocytes, microglia or endothelial cells in the optimized co-culture conditions. As expected, the B27 supplement was necessary to maintain the undifferentiated status of GSCs while FBS triggered their differentiation. Growth factors (EGF and FGF) appear to significantly alter the morphology of neurons and astrocytes and induce proliferation of microglia. They have been, therefore, omitted from the corresponding co-culture media. On the other hand, replacing FBS with B27 did not trigger significant morphological changes of primary glial and endothelial cells. Glutamine supplementation had significant effects on GSC and neurons (with low concentrations being toxic to the former and high concentrations to the latter) and, thus, had to be balanced for their co-cultures. N2 supplementation did not have major effects on the co-cultures. Furthermore, mRNA and protein levels of the representative markers for each recipient cell type have not changed under the corresponding co-culture conditions (Figure 3). Final compositions of the media supporting the co-cultures of GSCs with normal neurons, astrocytes, microglia, and brain endothelial cells are described in the Section “Materials and Methods.”

### Validation of Intercellular RNA Transfer in Co-cultures

**FIGURE 4:**
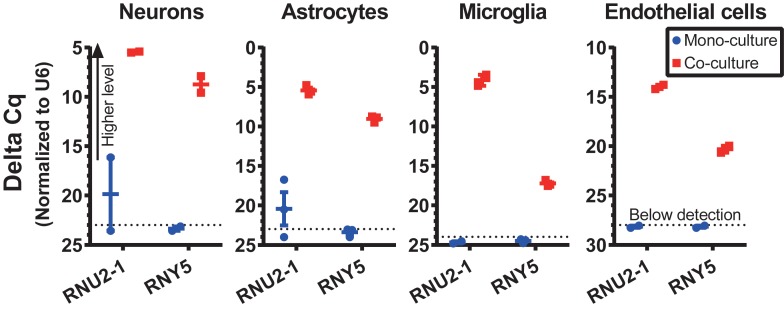
RNA transfer from human GSCs to mouse primary neurons, astrocytes, microglia, and endothelial cells was assessed for human-specific transcripts by qRT-PCR. *N* = 3 wells per culture condition, after 3 days in co-culture.

To validate a cross-talk between GSC and normal cells in the optimized co-culture conditions, human GSCs were co-cultured with various types of primary mouse brain cells, including neurons, astrocytes, microglia and endothelial cells, and the transfer of GSC-derived exRNA to the normal cells was assessed. The RNA was isolated from the recipient normal mouse cells after 3 days of co-culturing, and the levels of specific RNA markers in these cells were examined. We tested the transfer of snRNA and Y RNA, two classes of ncRNA abundantly released by GSCs (Wei et al., 2017). While human-specific RNAs such as RNU2-1 snRNA and RNY5 were undetectable in the monocultures of mouse primary microglia, they were strongly elevated in the microglia co-cultured with human GBM8 neurospheres (Figure 4). Similar results were observed for the recipient primary cultures of neurons, astrocytes, and endothelial cells (Figure 4). These experiments confirmed RNA transfer from human GSCs to the mouse recipient cells in co-cultures and thus demonstrated the application of this protocols for investigation of the intercellular communication between GSCs and normal cells of glioma microenvironment *in vitro*.

## Discussion

Although it is gaining increased attention in recent years, intercellular communication in the brain tumor microenvironment remains among the least investigated topics in the pathology of malignant gliomas. The impact of the intercellular interactions occurring in the brain on the glioma initiation, growth, and therapy resistance has not been thoroughly elucidated. Previous studies mostly focused on the secreted molecules that mediate communication, such as hormones, growth factors, cytokines, and chemokines. EVs and extravesicular RNP complexes carrying heterogeneous cargo (e.g., proteins, nucleic acids, and lipids) have been proposed as additional potent and more complex communication vehicles. Notably, the *in vitro* modeling of such complex interactions underlying the growth of intracranial tumors remains one of the major impediments in the field.

Most studies of the intercellular communication *in vitro* rely on the pulse supplementation of an experimental factor derived from the donor cells to the recipient cells. While such traditional strategy is merited for specific molecular agents/mediators (e.g., cytokines), it can be suboptimal for more complex mediators, such as EVs. It is currently unclear how to dose and time this type of experiments so that they mimic the physiology and dynamics of EV/RNP release and uptake; therefore, it is common to simply apply a pulse of hyper-concentrated preparations of EVs to the recipient cells. In addition, since any fresh media contain active ingredients that may cause false-positive results, designing suitable controls for such experiments is challenging (Wei et al., 2016; Tosar et al., 2017). Co-culturing diverse cell types may provide the better, more physiological approach for studying the intercellular communication among them. However, the caveat, especially concerning the difficult-to-culture primary and low passage cells of the brain and brain tumors, is their poor compatibility in culture. The cells of the CNS such as neurons, astrocytes, microglia, and brain derived endothelial cells are highly sensitive and require specific, distinct conditions for their growth *in vitro*. Optimized protocols for co-culturing these cells with glioma will advance the investigation of intercellular communication between brain tumors and brain microenvironment, mediated by EVs and other means (Broekman et al., 2018).

Here we optimized conditions for co-culturing four major types of normal brain cells with glioma-initiating cells so that they could be maintained together and retain their normal phenotypes for at least 6 days. This time frame is generally sufficient for a diverse set of molecular and phenotypic assays. In addition to revealing the impact of a broad EV population or the entire secretome, specific EV subclasses varying by their sizes (e.g., oncosomes, microvesicles, and exosomes) can be studied in such two-chamber systems separated by the membranes with different pore sizes. For example, modification of the assay to employ the inserts with either 0.2 μm and 20 nm pores will assess the relative contributions of EVs and RNPs.

In this study, we also propose several human-specific RNA markers that provide positive controls for RNA transfer studies and can be utilized for the studies of exRNA secretion and uptake mechanism. While the focus of exRNA field remains largely on miRNAs due to their enrichment in EVs and important regulatory potential, other classes of regulatory transcripts are abundantly released by various types of cells (Wei et al., 2017; Chiou et al., 2018; Driedonks and Nolte-’t Hoen, 2018; Hinger et al., 2018; Rimer et al., 2108; Elsemuller et al., 2019). The copy numbers of some non-miRNA transcripts in the extracellular space are significantly higher than those of even the most abundant miRNAs (Wei et al., 2017). While the biological impact of their intercellular transfer is largely unknown, the presented protocols may aid the corresponding investigation. Here we demonstrated the transfer of two classes of highly abundant ncRNA, snRNA, and Y RNA, from glioma cells to the normal cells of the brain. Overall, the established protocols are valuable for the investigation of intercellular communication between glioma brain tumor and its microenvironment, including but not limited to the EVs-mediated communication.

## Ethics Statement

All animal experiments have been approved by the Brigham and Women’s Hospital Institutional Animal Care and Use Committee.

## Author Contributions

AK conceived the study. ZW and AK designed the experiments and wrote the manuscript. ZW, SK, and REF performed the experiments. RR assisted with experiments. All authors revised and approved the manuscript.

## Conflict of Interest Statement

The authors declare that the research was conducted in the absence of any commercial or financial relationships that could be construed as a potential conflict of interest.

## Supplementary Material

TABLE S1 Tested medium compositions and their effects on cell growth and morphology. Optimal medium compositions for co-cultures are highlighted in red. *N* = at least two independent experiments/condition.
